# Structure–Function Relationships of Healthy and Osteoarthritic Human Tibial Cartilage: Experimental and Numerical Investigation

**DOI:** 10.1007/s10439-020-02559-0

**Published:** 2020-07-09

**Authors:** Mohammadhossein Ebrahimi, Mikael J. Turunen, Mikko A. Finnilä, Antti Joukainen, Heikki Kröger, Simo Saarakkala, Rami K. Korhonen, Petri Tanska

**Affiliations:** 1 Department of Applied Physics, University of Eastern Finland, POB 1627, 70211 Kuopio, Finland; 2 Research Unit of Medical Imaging, Physics and Technology, Faculty of Medicine, University of Oulu, Oulu, Finland; 3 SIBlabs, University of Eastern Finland, Kuopio, Finland; 4 Kuopio University Hospital, Kuopio, Finland; 5 Department of Diagnostic Radiology, Oulu University Hospital, Oulu, Finland

**Keywords:** Proteoglycan, Collagen fibril network, Fibril-reinforced poroelastic, Fourier transform infrared spectroscopy, Mechanical properties, Polarized light microscopy, Digital densitometry

## Abstract

Relationships between composition, structure and constituent-specific functional properties of human articular cartilage at different stages of osteoarthritis (OA) are poorly known. We established these relationships by comparison of elastic, viscoelastic and fibril-reinforced poroelastic mechanical properties with microscopic and spectroscopic analysis of structure and composition of healthy and osteoarthritic human tibial cartilage (*n* = 27). At a low frequency (0.005 Hz), proteoglycan content correlated negatively and collagen content correlated positively with the phase difference (i.e. tissue viscosity). At a high-frequency regime (> 0.05 Hz), proteoglycan content correlated negatively and collagen orientation angle correlated positively with the phase difference. Proteoglycans were lost in the early and advanced OA groups compared to the healthy group, while the superficial collagen orientation angle was greater only in the advanced OA group compared to the healthy group. Simultaneously, the initial fibril network modulus (fibril pretension) was smaller in the early and advanced OA groups compared to the healthy group. These findings suggest different mechanisms contribute to cartilage viscosity in low and high frequencies, and that the loss of superficial collagen pretension during early OA is due to lower tissue swelling (PG loss), while in advanced OA, both collagen disorganization and lower swelling modulate the collagen fibril pretension.

**Electronic supplementary material:**

The online version of this article (10.1007/s10439-020-02559-0) contains supplementary material, which is available to authorized users.

## Introduction

Articular cartilage provides the joint with smooth movements during locomotion. With its biphasic nature, cartilage is highly resilient and dissipative tissue. The fluid phase makes up approximately 70-80% of the wet weight of the tissue. The solid phase consists mainly of proteoglycans (PGs) (5–7% of the wet weight) and collagen fibril (mainly type II, 15–23% of the wet weight).^29^,^35^–^37^ The content, structure, and interactions between these main constituents control the mechanical behavior of cartilage. Cartilage responses to impacts and high-strain loads are mainly regulated by the collagen fibrils network and high interstitial fluid pressure. Under prolonged loading, fluid flows out of the tissue and PGs mainly control the equilibrium stiffness of the tissue.^37^,^51^

Osteoarthritis (OA) is a degenerative joint disease causing disabilities to large populations worldwide (e.g. approximately 9% of Americans^26^) and thus accounts for billions of dollars in the healthcare system. During the OA progression, tissue constituents experience substantial changes, such as loss of PGs and collagen, disorganization of collagen fibril network, and increased interstitial fluid content.^14^,^25^,^49^ These changes affect significantly the load-bearing capacity of the tissue.

Recent studies have employed animal models, such as steer,^47^ pony^50^ and most recently fetal ovine cartilage^6^ to investigate the relationships between structure, composition, and function of cartilage in OA. Animal models are very convenient to investigate complex biological processes promptly in well-controlled study cohorts (e.g. controlling age and gender). The onset and progression of different subtypes of OA, such as post-traumatic OA, can also be investigated in small^10^,^21^,^41^ and large animal models.^1^,^2^ Compared to small animal models, the use of large animal models enables studying the mechanisms of OA in a wider time frame, approaching the slow OA development in humans. Yet, understanding how native human tissue alters in OA provides novel insights into the disease, which is not possible to obtain directly from animal models. Investigations of human tissues can also elucidate how the animal model-based knowledge is transferrable to humans.

Natural differences between animal and human tissues have been reported in the literature.^3^,^9^ Structure–function relationships have been studied in human hip joint cartilage^31^ and human patellar cartilage.^23^ However, structure–function relationships in human tibial and femoral cartilage are still not well-known. This poses a challenge, for example, in a computational modeling of the knee joint. A better knowledge of structure–function relationships of cartilage will improve the accuracy of the models. In addition, structure–function relationships are important for *in vitro* studies to understand cell-tissue interactions.^8^ Therefore, a comprehensive investigation of the relationships between structure, composition and function of human tibial cartilage is essential.

The swelling pressure is known to be an important contributor to the mechanical behavior of cartilage. This has been investigated specifically through fibril-reinforced poroviscoelastic modeling of articular cartilage.^22^,^28^,^45^ The swelling pressure caused by the PG matrix modulates the collagen fibril reinforcement inside the cartilage.^55^ During transient conditions, collagen fibrils of the superficial zone, as well as fluid pressurization, carry a considerable amount of load. The loss of swelling and fluid pressurization may affect superficial collagen fibrils by diminishing their pre-tension, thus, impairing the contribution of collagen to the mechanical function of the tissue.^30^,^45^ However, none of the studies correlated the model-derived mechanical properties of articular cartilage with the structural and composition properties. The fibril-reinforced poroelastic (FRPE) material model incorporates the main constituents of cartilage and it can differentiate their contribution to the mechanical response. Combined with sample-specific finite element modeling, the FRPE material has shown the capability to accurately simulate experimentally derived data in dynamic and stress-relaxation tests.^24^ Fibril-reinforcement in the model, emphasized by high fluid pressurization, accounts for cartilage behavior particularly in short-term loading,^27^ while the non-fibrillar matrix is mainly responsible for the equilibrium response.

In our recent study,^15^ we observed that the severity of OA in human tibial cartilage was strongly associated with changes in the constituent-specific FRPE mechanical properties as well as elastic and viscoelastic properties. Comparing to the healthy tissue, we observed a great reduction in the initial fibril network modulus (related to the pretension of the collagen fibrils) and the non-fibrillar matrix modulus (related to the PG content). Moreover, elastic and viscoelastic mechanical properties exhibited negative and positive correlations, respectively, with the severity of OA.

In the present study, our objective was to investigate whether these previously observed alterations in the elastic, viscoelastic and constituent-specific FRPE mechanical properties at different stages of OA could be explained by changes in the structure and composition of cartilage. We hypothesized that:

1. the loss of initial fibril network modulus at different stages of OA is because of loss of PGs (loss of swelling pressure) and loss of collagen content
2. the PG content is the main contributor to the viscous response of cartilage (i.e. phase difference between sinusoidal displacement and force)

This study provides novel knowledge on structure–function relationships of human tibial cartilage at different stages of OA. This knowledge is also important to improve knee joint and multiscale modeling.

## Materials and Methods

### Sample Preparation, Mechanical Testing and Determination of the Elastic, Viscoelastic and Constituent Specific Material Parameters

As the biomechanical properties were reported in our previous study,^15^ a brief recap of sample preparation, mechanical testing and determination of the elastic, viscoelastic and constituent-specific (FRPE) parameters is provided in the supplementary material.

The process and use of the human tissue were approved by the National Authority for Medicolegal Affairs and the ethical committee of North Savo Hospital District (Ethical Permission Number 134/13.02.00/2015).

**Figure 1:**
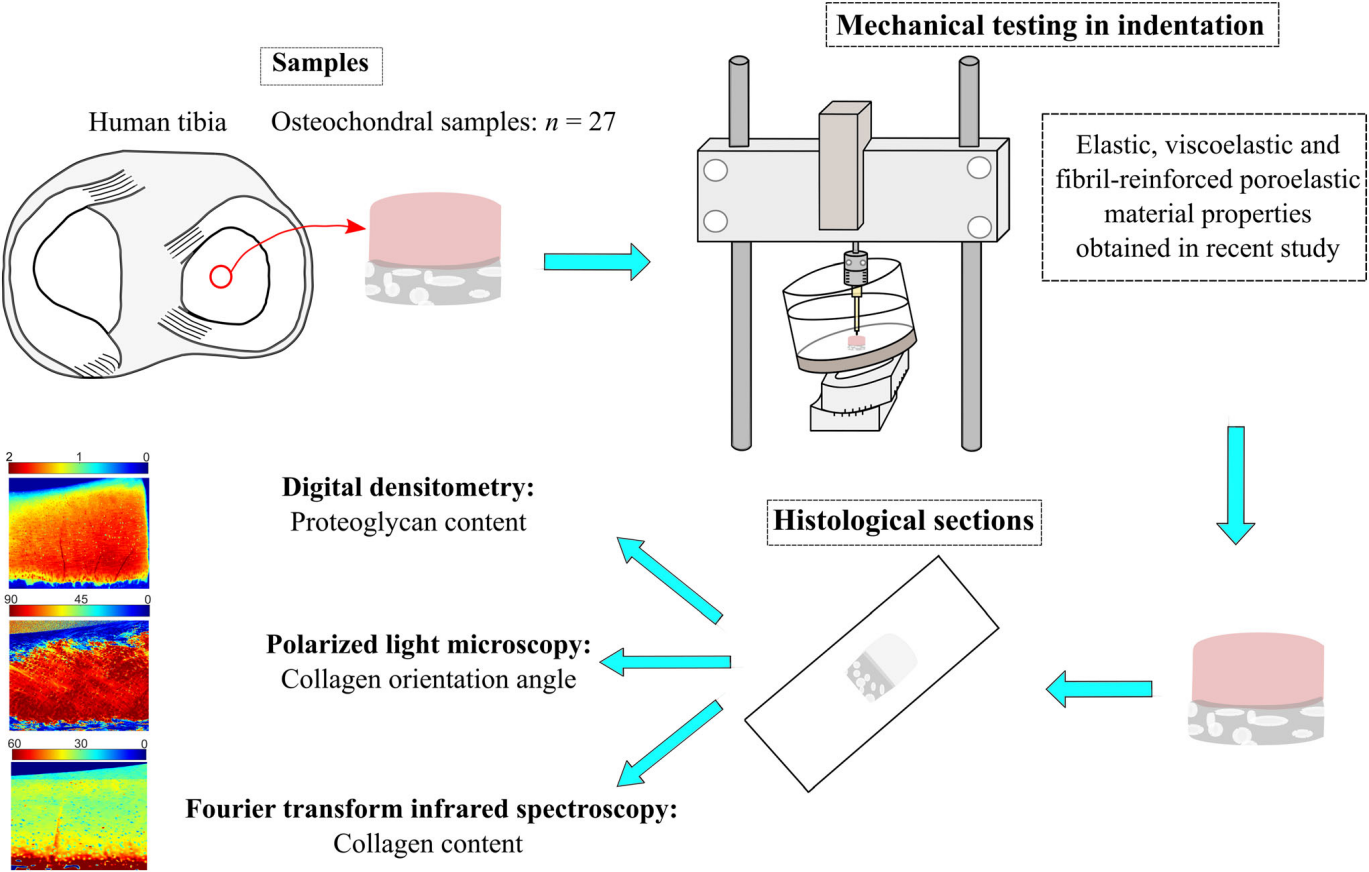
Workflow of the study. First, biomechanical measurements were conducted for the osteochondral samples obtained from tibia. Then, the samples were formalin-fixed, decalcified and embedded in paraffin tissue blocks. The blocks were sectioned and stained for OARSI grading. These steps were conducted in our previous study.^15^ In this study, the blocks underwent additional quantitative histological analyses including digital densitometry, polarized light microscopy and Fourier transform infrared spectroscopy.

In the current study, human osteochondral samples (*n* = 27) from tibial cartilage of 7 cadavers were processed to determine quantitative compositional and structural properties (Fig. 1). The OA severity of the samples was defined using the Osteoarthritis Research Society International (OARSI) histopathological grading system from Safranin-O sections. Samples were then grouped to healthy (OARSI grades 0–1), early OA (OARSI grades 2–3) and advanced OA (OARSI grade 4).

### Microscopical and Spectroscopical Analyses

In the current study, the samples were processed to determine quantitative compositional and structural properties using digital densitometry (DD), polarized light microscopy (PLM) and Fourier transform infrared spectroscopy (FTIR). Following the indentation tests, the samples were fixed in formalin and decalcified. Then, the samples were dehydrated in graded ethanol solutions and embedded in paraffin.^32^,^41^ Sections were prepared from the middle of the sample and perpendicular to the surface providing sections from the same area in which the biomechanical indentation test was performed. For DD measurements, 3 *µ*m thick sections were stained with Safranin-O. However, for PLM and FTIR measurements, 5 *µ*m thick sections were prepared. After cutting the sections, the paraffin was removed. The total number of sections for each set of microscopy/spectroscopy measurements was 81 (i.e. 3 adjacent sections per sample).

Digital densitometry was performed using a conventional light microscope (Nikon Microphot FXA, Nikon Co., Tokyo, Japan), CCD-camera (Hamamatsu ORCA-ER, Hamamatsu Photonics, Hamamatsu, Japan) and monochromator (*λ* = 492 ± 5 nm) to capture grayscale images of Safranin-O stained sections (pixel size = 3.09 × 3.09 *µ*m). The system was calibrated with filters (optical density values 0.0, 0.15, 0.3, 0.6, 1.0, 1.3, 1.6, 2.0, 2.6 and 3.0) (Schott, Mainz, Germany).^12^,^41^ Thus, optical density which is a quantitative estimator of the PG content of the samples was acquired. Three slices per sample were measured. Based on our analyses, the use of three sections provided consistent results within a sample (coefficient of variation < 0.1, see supplementary material). Three sections have also been used in several previous studies.^48^,^52^,^54^

A conventional light microscope (Nikon Diaphot TMD, Nikon, Inc., Shinagawa, Tokyo, Japan, pixel size = 2.53 × 2.53 *µ*m) equipped with Abrio PLM system (CRi, Inc., Woburn, MA, USA) was used to determine the orientation angle of the collagen fibrils.^19^,^46^ The Abrio system uses a circular polarizer consisting of two liquid crystal polarizers to assess the main orientation of the collagen fibrils in each pixel. Three sections per sample were measured.^48^,^52^,^54^

Agilent Cary 600 spectrometer coupled with Cary 610 FTIR microscope (Agilent Technologies, Santa Clara, CA, USA) was used to obtain spectral data for the analysis of the collagen content. Infrared light absorption spectrum with wavenumber ranging from 3800 to 750 cm^−1^ was collected pixel-by-pixel. To increase the signal to noise ratio, 8 scans per pixel were acquired. The spatial pixel size was 5.5 × 5.5 *µ*m and the spectral resolution was set to 4 cm^−1^. Following data acquisition, a constant baseline correction for the spectrum ranging from 2000 to 900 cm^−1^ was conducted. The Amide I region (1720–1595 cm^−1^) of the infrared spectrum was analyzed and used to estimate the collagen content.^48^,^49^ Three sections per sample were measured.^48^,^52^,^54^

The compositional and structural analyses were conducted in a 1.5 mm wide region of interest (averaging the pixel values in a horizontal direction) across the whole tissue depth using a custom-made code in Matlab software (v7.10.0, Mathworks Inc., MA, USA). Then, the three profiles obtained from three adjacent sections of each sample were averaged. Therefore, the depth-wise variations in the PG content, collagen content, and collagen orientation angle could be investigated in relation to the biomechanical and constituent-specific properties of cartilage.

### Statistical Analyses

To test our hypotheses, the measured structure and composition of human tibial cartilage were compared with the elastic, viscoelastic and constituent-specific FRPE material properties. The mechanical material properties were adopted from our previous study.^15^

The depth-wise changes in the cartilage structure and composition were compared between different OA groups using a linear mixed-effects model. Statistical power analyses were conducted to elucidate the power of statistical models and *post hoc* tests (see supplementary material).

Furthermore, a linear multivariable regression analysis was conducted to evaluate structure–function relationships. Analyses were conducted using IBM SPSS Statistics (version 25, IBM Corporation, Armonk, NY, USA; see supplementary materials).

## Results

**Figure 2:**
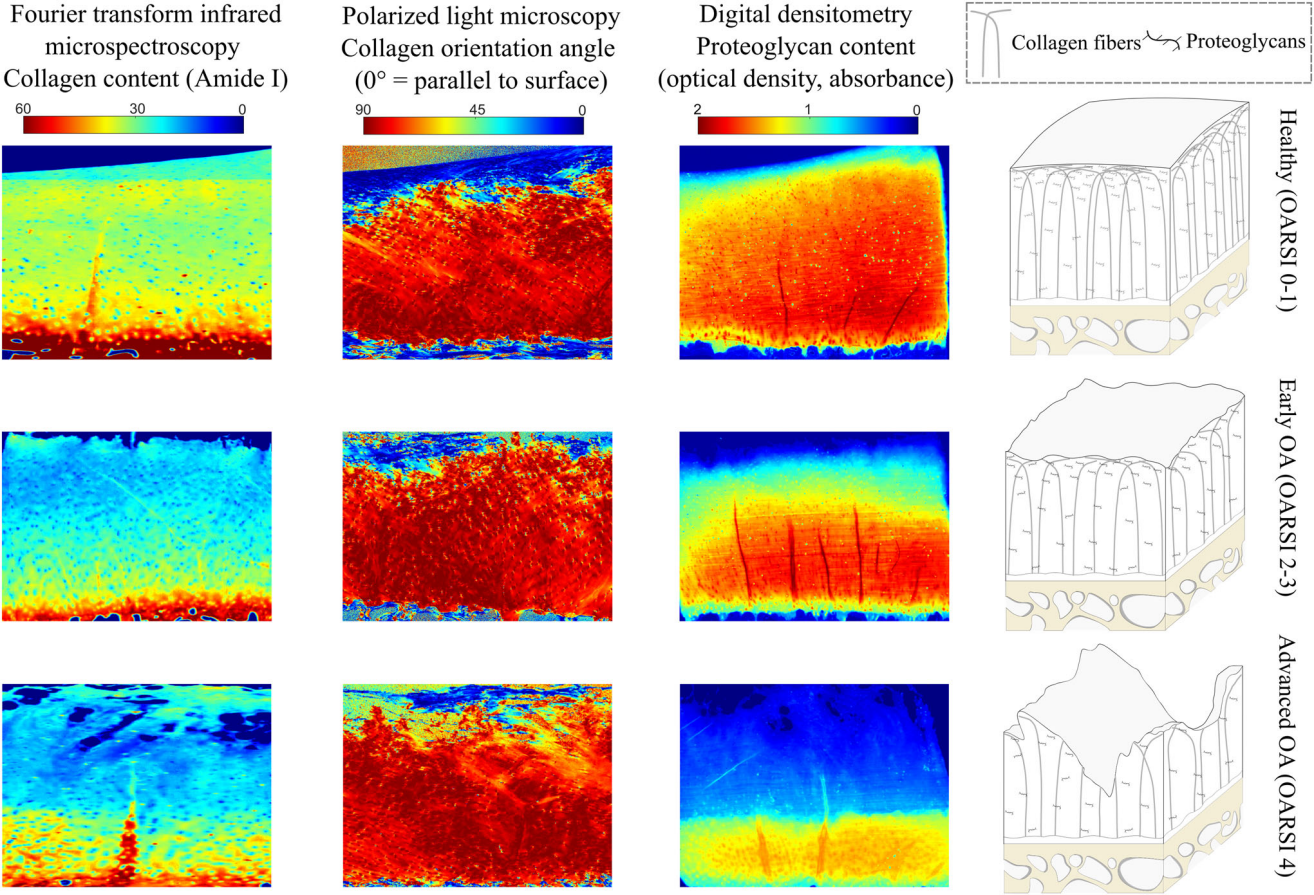
Representative images of collagen content, collagen orientation angle, and PG content in healthy (top row), early OA (middle row), and advanced OA (bottom row) cartilage.

Representative PG content, collagen content, and collagen orientation angle images from different OA groups are shown in Fig. 2.

### Depth-Wise Structural and Compositional Alterations of Human Tibial Cartilage at Different Stages of OA

**Figure 3:**
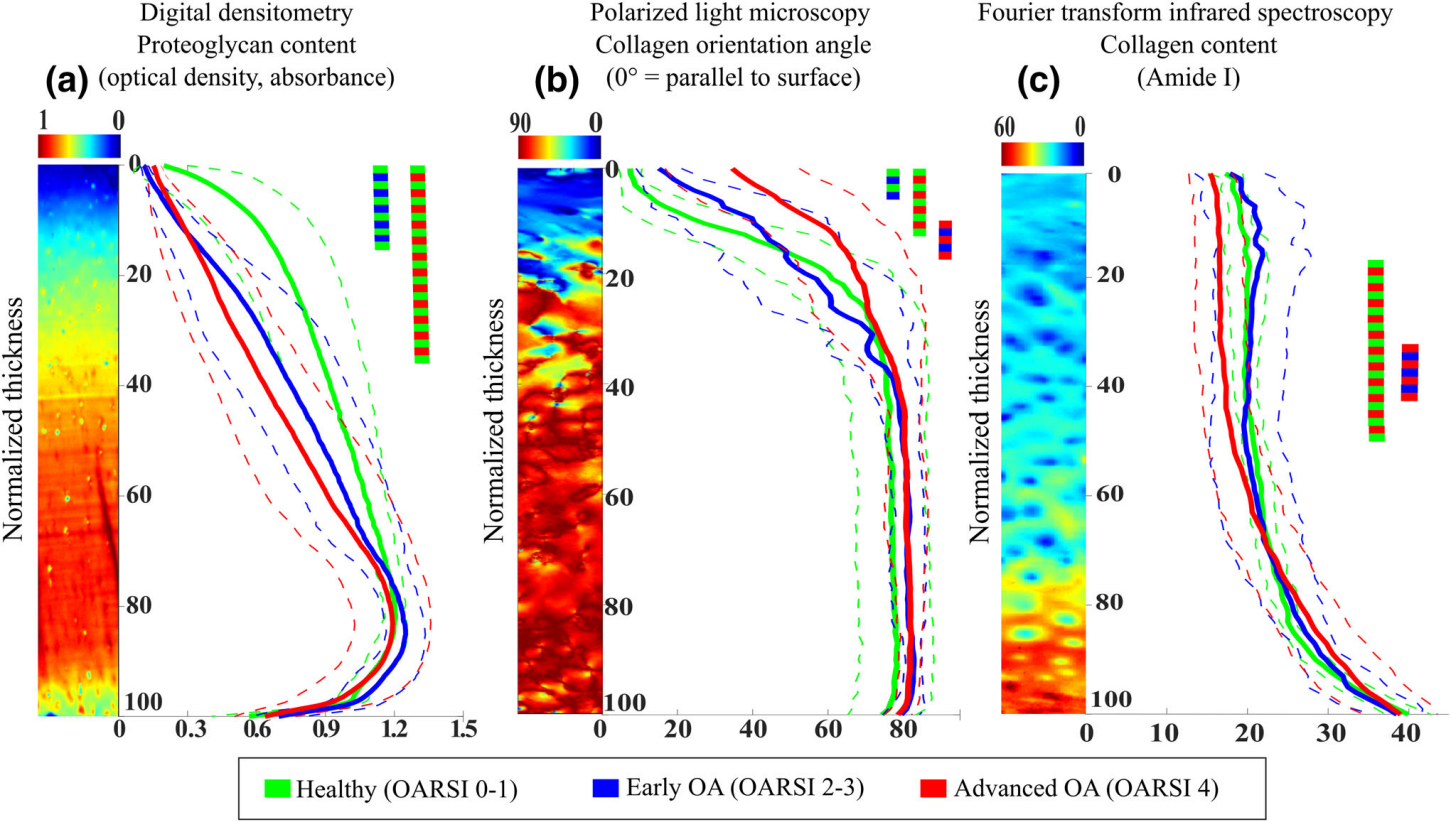
Depth-wise PG content, collagen orientation angle, and collagen content in healthy, early OA, and advanced OA cartilage. The solid line represents mean and the dotted line ± standard deviation. Colored bars indicate depth-wise regions with statistically significant differences between the groups, *p* < 0.05.

The depth-wise profiles of the composition/structure and point-by-point comparisons between the OA group profiles are shown in Fig. 3. Consistent with the changes in the equilibrium and non-fibrillar matrix modulus observed in our previous study,^15^ the PG loss occurred from 0 to 12% of the cartilage thickness in the *early OA* group compared to the *healthy* group. However, the PG loss in the *advanced OA* group compared to the *healthy* group progressed further, up to 37% of the cartilage thickness.

The superficial collagen fibrils were less parallel to the surface (i.e. greater orientation angle) in the *early OA* group compared to the *healthy* group in the superficial cartilage (from 0 to 4% of the thickness). In the *advanced OA* group, the collagen fibrils were less parallel to the surface up to 12% of the cartilage thickness compared to the *healthy* group. Moreover, the collagen orientation angle was greater in the *advanced OA* group compared to the *early OA* group at the depths ranging from 10 to 18% of the cartilage thickness.

The collagen content was not different between the *early OA* and *healthy* groups, while it was smaller in the *advanced OA* compared to the *healthy* group at the depths ranging from 17 to 52% of the cartilage thickness. Interestingly, the collagen content was also smaller at the depths ranging from 31 to 43% when the *advanced OA* group was compared to the *early OA* group.

#### Structure-Composition Correlation Analysis with the Elastic and Viscoelastic Mechanical Parameters

The PG content showed positive linear correlations with the equilibrium and initial instantaneous moduli at all tissue depths (Table 1, Supplementary Tables S1 and S2). The PG content also exhibited a positive linear correlation with the dynamic modulus at all depths, except at the depths of 0–5%. Importantly, the PG content analyzed up to 20% of the tissue thickness exhibited a negative linear correlation with the phase difference measured with 0.005 Hz sinusoidal loading frequency. However, the PG content of bulk cartilage exhibited a negative correlation with the phase difference at the higher frequencies (0.05–1 Hz). See supplementary material for the complete results.

**Table 1:** The standardized linear regression coefficients (β) of structural and compositional properties of the superficial (0–10% of tissue thickness) and bulk tissue with the mechanical parameters (***p < 0.001, **p < 0.01, *p < 0.05).

Dependent variable	Superficial cartilage (0–10%)				Bulk cartilage (0–100%)			
	Adjusted R^2^	PG content	Collagen orientation angle	Collagen content	Adjusted R^2^	PG content	Collagen orientation angle	Collagen content
$E_{\text{eq}}$	0.54	0.62***	− 0.34*	0.30	0.45	0.62***	− 0.36*	0.27
$E_{\text{inst}}^{0}$	0.42	0.52**	− 0.39*	0.01	0.28	0.41*	− 0.46*	0.17
$E_{\text{inst}}^{\varepsilon }$	0.10	0.05	− 0.38	0.18	0.31	0.36*	− 0.47*	0.40*
$E_{\text{dyn}}$	0.29–0.37	0.35*–0.36*	− 0.42*–− 0.46*	0.07–0.08	0.42–0.58	0.56***–0.57***	− 0.50**–− 0.52**	0.32–0.35
*θ*_*0.005*_	0.42	− 0.38*	− 0.08	0.58***	0.22	− 0.18	− 0.02	0.50*
*θ*_*i*_	0.10–0.24	− 0.09–− 0.23	0.30–0.41*	0.17–0.35	0.39–0.49	− 0.48**–− 0.59***	0.22–0.31	0.29–0.37

$E_{\text{eq}}$ equilibrium modulus, $E_{\text{inst}}^{0}$ initial instantaneous modulus, $E_{\text{inst}}^{\varepsilon }$ strain-dependent instantaneous modulus, $E_{\text{dyn}}$ dynamic moduli at frequencies 0.005, 0.05, 0.1, 0.25, 0.5, 0.625, 0.833 and 1 Hz, *θ*_0.005_ phase difference at 0.005 Hz, *θ*_*i*_ phase differences at frequencies 0.05, 0.1, 0.25, 0.5, 0.625, 0.833 and 1 Hz

The collagen orientation angle was negatively correlated with the equilibrium, initial instantaneous and dynamic moduli (Table 1, Supplementary Tables S1 and S2) at all tissue depths. Further, the collagen orientation angle exhibited a negative correlation with the strain-dependent instantaneous modulus at the depths of 0–20, 0–50 and 0–100% of the cartilage thickness. Furthermore, it exhibited no correlation with the phase difference at 0.005 Hz. Interestingly, the collagen orientation angle of the superficial cartilage was positively correlated with the phase difference at higher frequencies (0.05, 0.1, 0.5, 0.833 and 1 Hz, Supplementary Table S3).

The collagen content exhibited a positive correlation with the phase difference at 0.005 Hz at all depths (Table 1, Supplementary Tables S1 and S2). Further, the collagen content exhibited a positive correlation with the strain-dependent instantaneous modulus only when the whole tissue was analyzed.

#### Structure-Composition Correlation Analysis with the Constituent-Specific Mechanical Parameters

The multivariable regression analyses for the model-derived material parameters are shown in Table 2 for the superficial (0–10%) and bulk tissue (0–100%). The analyses for the other depths (0–5, 0–15, 0–20 and 0–50%) and structure–function scatter plots are presented in the supplementary material.

**Table 2:** Structure-function relationships between the structural, compositional and FRPE material properties in the superficial (0–10% of the normalized tissue thickness) and bulk cartilage using multivariable linear regression (**p < 0.01, *p < 0.05).

Dependent variable	Standardized regression coefficient β							
	Superficial cartilage (0–10%)				Bulk cartilage (0–100%)			
	Adjusted R^2^	PG content	Collagen orientation angle	Collagen content	Adjusted R^2^	PG content	Collagen orientation angle	Collagen content
$E_{\text{f}}^{0}$	0.31	0.46**	− 0.32	0.08	0.30	0.34*	− 0.51**	0.32
$E_{\text{f}}^{\varepsilon }$	0.05	− 0.22	− 0.29	0.14	0.08	0.31	− 0.29	0.21
$E_{\text{nf}}$	0.37	0.51**	− 0.32	0.05	0.23	0.46*	− 0.31	0.26
$k_{0}$	0.12	− 0.30	0.06	0.16	0.08	− 0.34	− 0.09	0.26
$M$	0.04	− 0.10	− 0.23	0.10	0.29	− 0.27	− 0.37*	0.46**

These material parameters were explained and derived in Ref. 15

$E_{\text{f}}^{0}$ initial fibril network modulus, $E_{\text{f}}^{\varepsilon }$ strain-dependent fibril network modulus, $E_{\text{nf}}$ non-fibrillar matrix modulus,$k_{0}$ initial permeability, $M$ permeability strain-dependency coefficient

The PG content exhibited a positive linear correlation with the initial fibril network modulus and the non-fibrillar matrix modulus at all tissue depths (Table 2 and Supplementary Tables S1 and S2). The PG content did not exhibit any linear correlation with the initial permeability, but exhibited a negative monotonic rank correlation with the initial permeability at all tissue depths (Table 3). The PG content did not exhibit any linear or rank correlations with the strain-dependent fibril network modulus or the permeability strain-dependency coefficient.

**Table 3:** Depth-wise Spearman’s monotonic rank correlation analyses between the structural, compositional and FRPE material properties (**p < 0.01, *p < 0.05).

Parameter	Tissue depth (%)	$E_{\text{f}}^{0}$	$E_{\text{f}}^{\varepsilon }$	$E_{\text{nf}}$	$k_{0}$	$M$
PG content	0–5	ns.	ns.	ns.	− 0.39*	ns.
	0–10	ns.	ns.	ns.	− 0.46*	ns.
	0–15	ns.	ns.	ns.	− 0.45*	ns.
	0–20	ns.	ns.	ns.	− 0.47*	ns.
	0–50	ns.	ns.	0.43*	− 0.44*	ns.
	0–100	ns.	ns.	0.41*	− 0.39*	ns.
Collagen orientation angle	0–5	ns.	ns.	− 0.54**	0.46*	ns.
	0–10	ns.	ns.	− 0.52**	0.45*	ns.
	0–15	ns.	− 0.41*	− 0.51**	0.39*	ns.
	0–20	ns.	− 0.44*	− 0.41*	ns.	ns.
	0–50	ns.	− 0.42*	− 0.40*	ns.	ns.
	0–100	ns.	− 0.39*	ns.	ns.	ns.
Collagen content	0–5	ns.	ns.	ns.	ns.	ns.
	0–10	ns.	ns.	ns.	ns.	ns.
	0–15	ns.	ns.	ns.	ns.	ns.
	0–20	ns.	ns.	ns.	ns.	ns.
	0–50	ns.	ns.	ns.	ns.	0.40*
	0–100	ns.	ns.	ns.	ns.	0.38*

$E_{\text{f}}^{0}$ initial fibril network modulus, $E_{\text{f}}^{\varepsilon }$ strain-dependent fibril network modulus, $E_{\text{nf}}$ non-fibrillar matrix modulus,$k_{0}$ initial permeability, $M$ permeability strain-dependency coefficient, *ns.* not significant

The collagen orientation angle exhibited a negative linear correlation with the initial fibril network modulus at the depths of 0–20, 0–50 and 0–100% (Table 2 and Supplementary Tables S1 and S2). The collagen orientation angle did not show a linear correlation with the initial permeability, but showed a positive rank correlation with the initial permeability at up to 15% of the tissue thickness (Table 3). Interestingly, while the collagen orientation angle did not exhibit linear or rank correlations with the strain-dependent fibril network modulus in the superficial tissue (analyzed from the surface up to 10% of the tissue thickness), there was a negative monotonic rank correlation between these parameters when deeper parts of the tissue were included in the analysis. Further, the collagen orientation angle exhibited a negative linear correlation with the permeability strain-dependency coefficient only when the bulk tissue was analyzed.

The collagen content exhibited both linear and rank correlations with the permeability strain-dependency coefficient when the analysis was conducted at the depths of 0–50 and 0–100% (Table 2 and Supplementary Table S2).

#### Changes in the Structure and Function of Cartilage at Different Stages of OA

**Figure 4:**
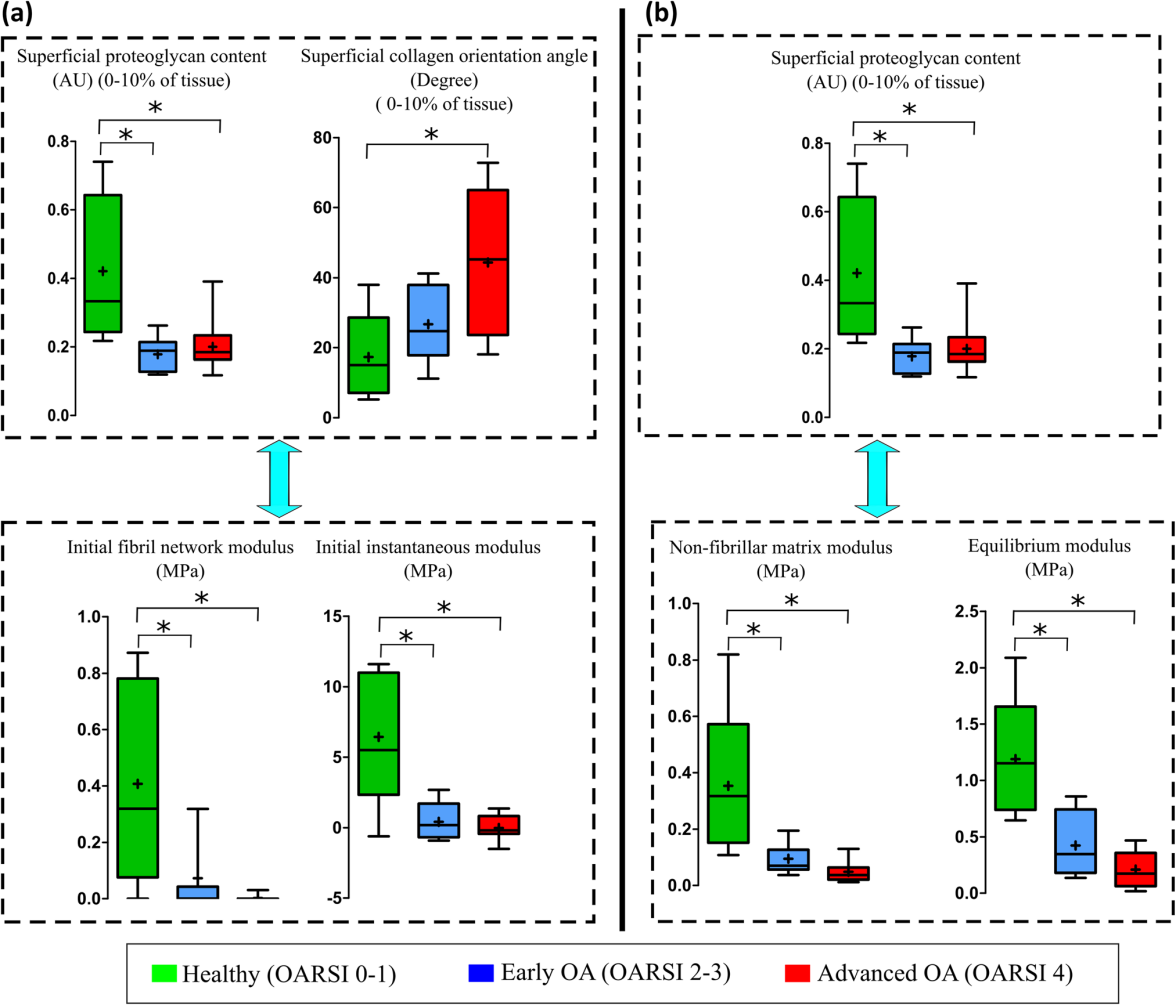
The summary of the observed changes in the structure and function of cartilage at different stages of OA. The results suggest that (**a**) the loss of collagen pretension (= the initial fibril network modulus) at different stages of OA (increasing OARSI grade) is explained by the changes in the composition (PG content) and structure (collagen orientation) of the superficial cartilage. In *early OA* cartilage, the loss of collagen pretension is mainly regulated by the PG content, while in *advanced OA* cartilage, it is regulated by both the PG content and collagen disorganization. The results also suggest that (**b**) the smaller non-fibrillar matrix and equilibrium moduli of cartilage are explained by the loss of PG content of the superficial cartilage in *early* and *advanced OA* cartilage compared to *healthy* cartilage, *p < 0.05.

The PG content was smaller in the *early* and *advanced OA* groups compared to the *healthy* group. However, the collagen disorganization (as characterized by an increase in the collagen orientation angle) occurred only in the *advanced OA* group compared to the *healthy* group. Previously, we had observed that the initial fibril network modulus and the initial instantaneous modulus were lower in the *early* and *advanced OA* groups compared to the *healthy* group (Fig. 4a).^15^

Furthermore, we had also previously observed that the non-fibrillar matrix modulus and equilibrium modulus were lower in the *early* and *advanced OA* groups compared to the *healthy* group^15^ (Fig. 4b). In the current study, the PG content showed similar changes.

## Discussion

In the present study, healthy and osteoarthritic human tibial cartilage samples were characterized by investigating the depth-wise structure–function relationships. Specifically, the model-derived constituent-specific material properties and “traditional” elastic and viscoelastic biomechanical properties were compared with quantitative depth-wise structural and compositional properties (i.e. PG content, collagen content, and collagen orientation angle). By characterizing these, we obtained new insights on how each cartilage constituent contributes to the mechanical response of the tissue at different stages of OA. This is the first study to establish structure–function relationships in human tibial cartilage at different stages of osteoarthritis.

### Depth-Wise Structural and Compositional Alterations in Human Cartilage at Different Stages of OA

We used different microscopic and spectroscopic measurements to characterize the depth-wise structure and composition (distribution of collagen fibrils and PGs). The PG content of osteoarthritic tibial cartilage was lower at the early stages of OA when compared to the healthy tissue. The loss of superficial PG content is a well-accepted sign of early OA, shown e.g. in rabbit models of OA^5^,^17^ and humans.^49^ Based on earlier studies and the present results, the PG loss of tibial cartilage progresses to deeper layers of cartilage at the later stages of OA.

The collagen content, on the other hand, showed no signs of changes in early OA. Consistent with our results, studies using a rabbit model of early OA^5^,^33^,^53^ and human cartilage^38^,^49^ showed no significant changes in the collagen content during early OA. However, some earlier rabbit OA model studies have contradictorily reported an increase in the collagen content in the deep cartilage near the bone-cartilage interface during early stages OA,^17^,^32^ presumably due to the up-regulation of collagen synthesis. The difference to our study might be due to different species (human vs. rabbit) and different type of OA (secondary OA vs. primary OA). In the current study, the collagen content showed signs of a decrease at the late stage of OA in the middle and deep cartilage layers. Consistent with this finding, the collagen content of human patellar cartilage has been suggested to decrease only at the later stages of OA.^49^

PLM has been widely used to determine the orientation angle and organization of collagen fibrils.^4^,^39^,^49^ In this study, PLM measurements indicated that collagen fibrils disorganize at the superficial cartilage (0–4%) in early OA. Fibrillation of the cartilage surface has been reported in the literature as an early sign of OA.^4^ In advanced OA, the disorganization of the collagen fibrils extended deeper in the tissue and was evident up to 12% of the tibial cartilage thickness. However, as we do not know the initial state of the tissue (i.e. we do not have follow-up), we cannot certainly state what mechanism causes the change in the collagen orientation or what kind of initial structure may be more prone to orientation alterations when OA progresses. There might be several mechanisms behind these changes, e.g., (1) change in the PG content could lead to altered tissue loading and disruption of the normal architectural organization of the collagen network^27^,^55^ (2) mechanical failure of collagen fibrils could lead to altered architecture and mechanical properties of the fibrillar network.^49^,^54^

### Associations Between the Mechanical Material Properties, Structure and Composition

In our previous study,^15^ we reported that the initial fibril network modulus (i.e. pretension of the collagen fibrils in the superficial cartilage) decreased as OA progressed. In the present study, we observed that the initial fibril network modulus (and similarly the initial instantaneous modulus) correlated positively with the PG content and negatively with the collagen orientation, but it did not correlate with the collagen content. This latter observation is partly against our original hypothesis. This can be since we did not observe differences in the superficial collagen content (presumably the region related to the initial fibril network modulus) between OA and healthy groups. Thus, the correlation analysis included a too homogeneous group of samples.

The group-wise comparison revealed that the PG content was lower in the early and advanced OA groups compared to the healthy group. Simultaneously, the collagen fibril pretension was also lower in the early and advanced OA groups compared to the healthy group. We hypothesize two potential mechanisms for these observations. First, when negatively charged PGs are depleted, the swelling pressure of the tissue caused by Donnan swelling will decrease. Therefore, the pretension of collagen fibrils will decrease. This is reflected by the smaller initial fibril network modulus in our model in the OA groups compared to the healthy group. Second, the collagen network is initially damaged which is observed as fibrillation of the tissue and reduction of the initial fibril network modulus. This may lead to the transportation of PGs through the loosened fibrillar network, resulting in a loss of fixed charge density and swelling pressure of the tissue. PG leakage may also be accelerated by fluid flow when cartilage is compressed.^42^

The collagen orientation angle in the superficial tissue increased progressively with the stage of OA, but on average at 10% of the tissue thickness, the collagen orientation was disorganized (i.e. orientation angle was greater) only in the advanced OA group compared to the healthy group. Together with earlier alterations in the initial fibril network modulus and PG content (see above and Fig. 4), this result suggests that the loss of the collagen pretension in the superficial tissue during early OA is mainly due to the lower tissue swelling (caused by the loss of PGs) rather than collagen disorganization. However, the loss of collagen fibril pretension in advanced OA is suggested to be regulated by both collagen disorganization and lower tissue swelling.

### Associations Between Viscosity (Phase Difference), Structure and Composition

Interestingly, at a very low frequency (0.005 Hz), we observed (1) a negative correlation between the PG content and the phase difference (viscosity) and (2) a positive correlation between the collagen content and the phase difference. This could imply that cartilage with greater collagen content and smaller PG content could be more viscous (i.e. less elastic). The fluid pressurization and flow (i.e. poroelasticity) may also contribute less to the apparent viscoelastic mechanical response as fluid pressure is reduced at low loading frequencies^40^ (see supplementary material for additional discussion and Figure S3). Thus, the contribution of collagen fibrils to apparent viscoelastic mechanical response is presumably more pronounced compared to fluid flow (poroelasticity) at low loading frequencies (e.g. 0.005 Hz). This may also include the interactions between PGs and collagen fibrils and/or electrostatic repulsion between PGs themselves and these interactions can also be altered when tissue degenerates. Further, a recent study also suggested that the viscoelastic energy dissipation could be the dominant factor at lower frequencies and poroelastic energy dissipation could be the dominant factor at higher frequencies in soft connective tissues (skin and tendon) in close to micrometer scale.^40^ However, caution is advised when relating our results and the previous study as the length scales are substantially different (~ 1 mm vs ~ 1 μm as poroelasticity is length scale dependent i.e. we might still measure poroelastic contribution in our measurements) and different tissues were characterized.

At higher frequencies (ranging from 0.05 to 1 Hz), the collagen content did not correlate with the phase difference. Instead, we observed (1) a positive correlation between the collagen orientation angle and the phase difference and (2) a negative correlation between the PG content and the phase difference. The first observation would suggest that cartilage with a less organized structure could dissipate more energy (as it is more viscous) at higher frequencies. Further, as in the very low loading frequency case, our data suggest that at high frequencies the smaller PG content also increases viscous mechanical behavior. However, the viscous behavior at high loading frequencies is most likely controlled by the poroelasticity. We also observed that greater collagen disorganization and smaller PG content were associated with an increase in cartilage permeability at the superficial cartilage. This suggests that the loss of the PGs and the disorganization of collagen fibrils facilitate the fluid flow in cartilage. This finding was partly against our original hypothesis in which we stated that viscous properties of cartilage are controlled only by the PG content. Finally, we also observed that the phase difference and dynamic modulus reached a plateau at around 0.1 Hz, suggesting that the dynamic properties would be similar at frequencies higher than 1 Hz.

Based on our observations and the related conclusions it seems that fluid flow-independent collagen viscoelasticity may be more dominant on the apparent viscous response of the tissue at low frequencies while the fluid flow-dependent viscoelasticity (i.e. poroelasticity) is dominant on the apparent viscous response at higher frequencies. However, fluid flow-dependent viscoelasticity may still contribute to the viscous response at lower frequencies to some extent.

The positive rank correlation between the permeability strain-dependency coefficient and the collagen content suggests that higher content (as collagen is presumably more densely packed) impair the fluid flow at high compressive strains, thus reducing permeability as a function of strain. This can be the case especially for *healthy* samples, which had higher collagen content at middle and deep layers compared to *advanced OA* samples. This finding is also consistent with a recent human hip joint cartilage study^31^ which reported a similar positive correlation between the permeability strain-dependency coefficient and collagen content.

### Limitations and Applications

Generally, cartilage becomes thinner in the advanced stages of OA.^16^ On the other hand, for early OA animal models, investigators have also reported an increase in tissue thickness due to the disorganization of the collagen network and cartilage swelling.^7^,^11^,^43^ This thickness difference could potentially affect depth-wise analysis when comparing structural and compositional properties between OA groups as a function of the cartilage thickness. However, in our study, the sample thicknesses were not statistically different between the groups^15^ (*p* value = 0.99 for linear mixed model). A recent study also showed that the thickness of human tibial cartilage was not associated with the severity of cartilage degeneration (OARSI grades between 0 and 4).^13^ Cartilage thinning is associated with very late stages of OA, e.g. OARSI 5-6.^44^ Moreover, biomechanical testing and analysis of tissue composition and structure were consistently performed to the normalized tissue thickness. Therefore, the comparison between the structural, compositional and functional properties in this study should be plausible.

To thoroughly characterize the structure–function relations during the progression of human OA, the initial condition of cartilage must be known and the OA progression of that sample must be followed. This could be possible by *in vivo* imaging, such as MRI, but then a detailed and high-resolution characterization of tissue structure and function, as done here, would not be possible.

Another limitation of the current study is related to the number of samples in different OA groups. For instance, there were 17 samples in the *advanced OA* group against only 5 samples in the *healthy* group. This was since the samples were obtained from relatively old cadavers (age 71.4 ± 5.2 years). Due to this our sample pool might be too homogenous which is partly seen in our concentrated data points in the scatter plots (see supplementary material Figure S2). To elucidate the strength of our statistical conclusions, we conducted an additional power analysis for the data. We observed that the linear mixed-effects model had a very high power (ranging from 0.94 to 0.99, Supplementary Material Table S4). In addition, the observed power for identifying the group-wise differences was also relatively high (ranging from 0.47 to 0.97) for the significantly different groups (Supplementary Material Table S5).

Recent studies have developed sophisticated knee joint models for the prediction of OA progression.^18^,^34^ However, none of the earlier models have included realistic human cartilage properties. The knowledge from the current paper can improve the accuracy of those models. In addition, this knowledge is important for multiscale studies investigating cell-tissue interactions at different tissue depths.^8^

Further, a better understanding of the associations between the mechanical properties and biochemical content and structure within native human cartilage may help to develop methods for non-destructive evaluation of cartilage.^20^ In the future, we aim to apply computational methods to estimate the function of cartilage based on the composition and structure, thus, the direct measurement of tissue function (e.g. indentation) would not be needed.

### Conclusion

In conclusion, the present study provides novel information about the complex structure–function relationships in human tibial cartilage. The results suggest that the loss of collagen fibrils pretension in the superficial cartilage is controlled by the loss of PG content (i.e. swelling pressure) at early OA and by the combination of the loss of PG content and greater disorganization of collagen fibrils at advanced/late OA. Furthermore, the results suggest that different mechanisms (fluid flow-independent collagen viscoelasticity and poroelasticity) may contribute to cartilage viscosity in low and high frequencies.

## Electronic supplementary material

Below is the link to the electronic supplementary material.

Supplementary material 1 (PDF 2231 kb)
