# Supplementary material for: Structure–Function Relationships of Healthy and Osteoarthritic Human Tibial Cartilage: Experimental and Numerical Investigation
Source: Ann Biomed Eng. 2020 Jul 9;48(12):2887–900. doi: 10.1007/s10439-020-02559-0 (PMC7723942; doi:10.1007/s10439-020-02559-0)
Supplement: Supplementary file 1 — Supplementary material 1 (PDF 2231 kb) [file 10439_2020_2559_MOESM1_ESM.pdf]

Supplementary materials for:

Structure-function relationships of healthy and osteoarthritic human tibial cartilage – experimental and numerical investigation

Mohammadhossein Ebrahimi[1,2*], Mikael J. Turunen[1,3], Mikko A. Finnilä[2], Antti Joukainen[4], Heikki Kröger[4], Simo Saarakkala[2,5], Rami K. Korhonen[1], Petri Tanska[1]

[1]Department of Applied Physics, University of Eastern Finland, Kuopio, Finland; [2]Research Unit of Medical Imaging, Physics and Technology, Faculty of Medicine, University of Oulu, Oulu, Finland; [3]SIBlabs, University of Eastern Finland, Kuopio, Finland; [4]Kuopio University Hospital, Kuopio, Finland; [5]Department of Diagnostic Radiology, Oulu University Hospital, Oulu, Finland

***Corresponding author:**
Mohammadhossein Ebrahimi
Department of Applied Physics
University of Eastern Finland
POB 1627, FI-70211 Kuopio
Finland
Tel. +358-449691559
E-mail: mohammadhossein.ebrahimi@uef.fi ; ebrahimi.bioengineering@gmail.com

Materials and Methods

Sample preparation, mechanical testing and determination of the elastic, viscoelastic and constituent specific material parameters

These measurements were conducted in our previous study [3], thus, only a brief recap is given here. Osteochondral samples (n = 27) were harvested from tibial cartilage of seven cadavers (age 71.4 ± 5.2 years, range 68–79 years, 6 males and 1 female) and kept in PBS at -23 ^{o}C until thawed for mechanical indentation tests. The isotonic PBS with enzymatic inhibitors was used to mimic the natural environment of cartilage (pH 7.4, 300mOsm), similar to numerous earlier studies [6,8,9,14]. It contained ethylenediamine tetraacetic acid disodium salt (1.86 g/L, EDTA VWR International, Radnor, PA, USA), and enzymatic inhibitors of benzamidine hydrochloride hydrate (0.78 g/L, Sigma-Aldrich Co., St. Louis, MO, USA) [6,14].

Before the actual test, a small pre-load of 12.5 kPa was applied to ensure proper contact between the indenter and sample. This preload was selected based on a previous studies [5,8]. It was also essential to ensure that the preload does not cause considerable deformation to softer samples (10-100 µm deformation was observed depending on the sample thickness and stiffness). We then performed a 4-step stress-relaxation protocol in indentation with 5% strain and 15-minute relaxation time at each step. In the compression phase of each stress-relaxation step, a strain rate of 100%/s was applied to obtain the maximum force where fluid pressure is high and altered collagen-fluid interaction can be detected. The same strain rate has also been used in previous studies [8,10]. It also is in the strain rate region that known to occur during daily activities [1]. The multi-step protocol was chosen to investigate non-linear properties of cartilage, while less than 20% total strain should not cause damage to the tissue [5,8]. The relaxation time (i.e. 15 minutes) was based on our preliminary test as well as previous studies [2,4]. Preliminary test showed that 15-minute relaxation time is adequate to let our sample reach to equilibrium phase. The stress-relaxation protocol was followed by a sinusoidal test (i.e. the total tissue strain was 20%) with a 2% strain amplitude (from the remaining thickness) at frequencies of 0.005, 0.05, 0.1, 0.25, 0.5, 0.625, 0.833 and 1 Hz. Based on earlier studies [7,13], this frequency range was assumed to show changes in the dynamic modulus and phase difference, especially at low frequencies.

Accordingly, dynamic moduli at each frequency (E_{dyn}), phase differences at each frequency (θ), equilibrium modulus (E_{eq}), initial instantaneous modulus (E_{inst}^{0}) and strain-dependent instantaneous modulus ($E_{\mathrm{inst}}^{\varepsilon}$) were calculated. A fibril-reinforced poroelastic (FRPE) finite element model was used to determine the non-fibrillar matrix modulus (E_{nf}), initial (E_{f}^{0}) and strain-dependent ($E_{\mathrm{f}}^{\varepsilon}$) fibril network moduli, initial permeability (k_0) and permeability strain-dependency coefficient (M). The details of the FRPE material model and determination of the FRPE material properties of the samples are presented in our previous study [3].

Justification for choosing three slices

Based on our preliminary analysis we observed that 3 sections give a consistent result within a sample (standard deviation normalized by mean < 0.1). The image below is to show the average and standard deviation profiles of three slices of a randomly selected sample. As can be seen clearly from this figure, the standard deviations are quite small (standard deviation normalized by mean < 0.1), supporting that three slices should be sufficient to provide a meaningful average. We can see the same behavior in all samples.

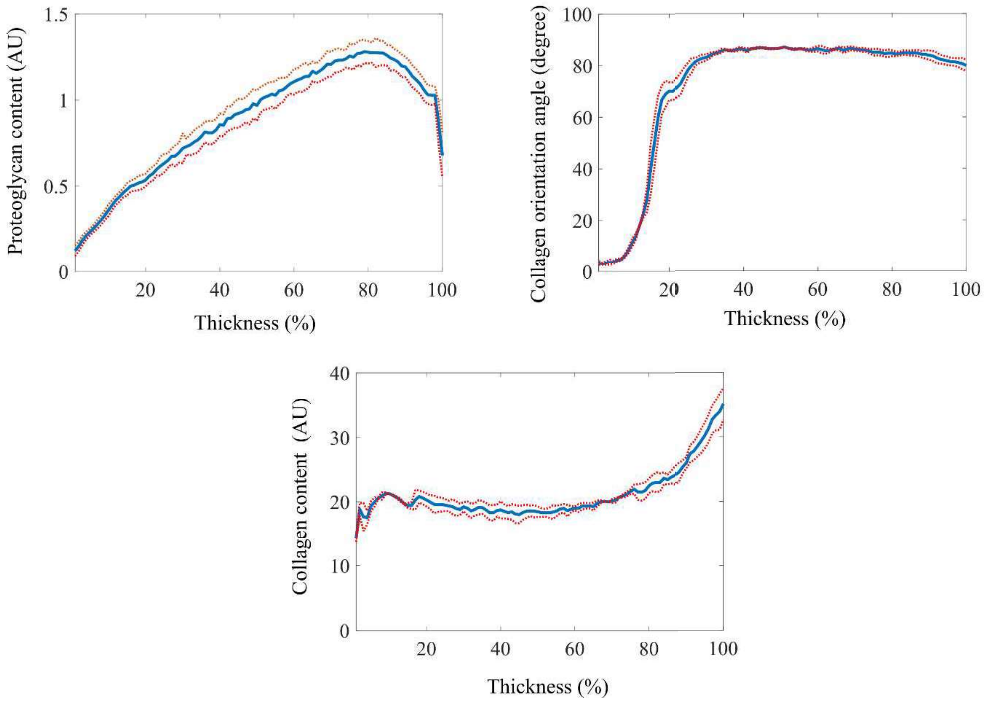

Figure S1: The average profile (solid blue line) and standard deviation (dashed red lines) of proteoglycan content, collagen orientation angle and collagen content of three slices of a randomly selected sample (OARSI = 1).

Statistical analyses

First, the thickness of the tissue was normalized to conduct the analyses in a depth-wise manner (0 = cartilage surface and 1 = cartilage-bone interface). Then, the depth-wise changes in the cartilage structure and composition were compared between different OA groups using a linear mixed model. By a proper selection of random effects, this method can account for the possible interrelations between the samples, which may occur since some samples were harvested from the same cadaver. In the model, subjects in each group were selected as a random effect and the OA group (*healthy*, *early OA* or *advanced OA*) was set as a fixed variable. Bonferroni adjustment was conducted for multiple comparisons to obtain conservative estimates for statistically significant differences between the groups. In addition, a statistical power analysis was conducted (OpenEpi, V3.01, Open Source Epidemiologic Statistics for Public Health, www.OpenEpi.com) to elucidate the strength of the statistical conclusions and the effect of the sample size.

Furthermore, a linear multivariable regression analysis was conducted to evaluate structure-function relationships. In this analysis, the elastic, viscoelastic, as well as the constituent-specific (FRPE) material parameters of cartilage, were evaluated as dependent variables and PG content, collagen orientation angle and collagen content as predictors. Finally, Spearman's rank correlation analysis was conducted to evaluate if there is a monotonic rank correlation between the constituent-specific material parameters and structure and composition. This was justified as the functional properties may not be linearly dependent on the composition and structure. In all analyses, $p < 0.05$ was considered statistically significant. Statistical analyses were performed by IBM SPSS Statistics (version 25, IBM Corporation, Armonk, NY, USA).

Results

The scatter plots of model-derived material parameters and structure/composition:

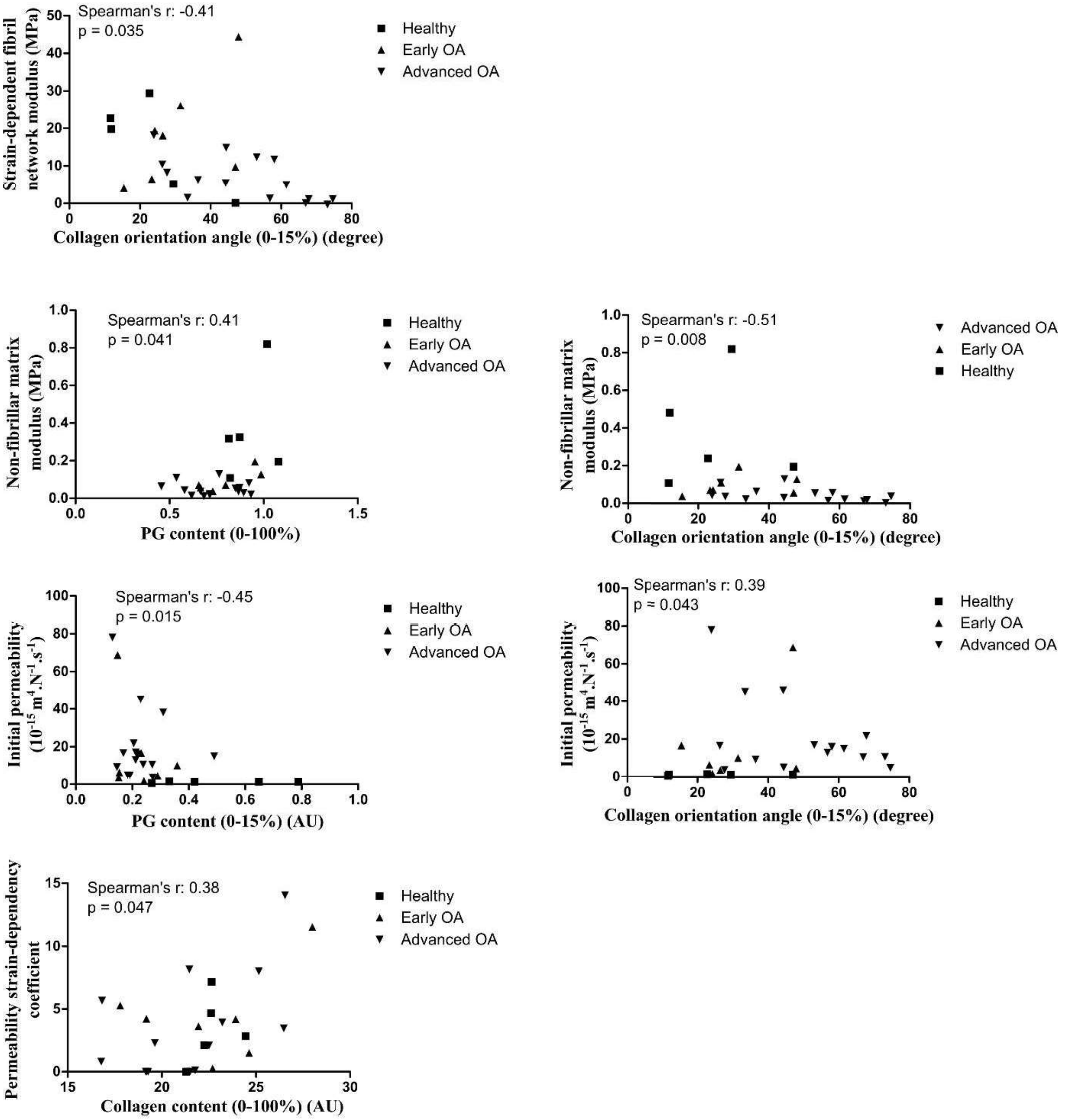

Figure S2: The linear and monotonic correlation coefficient of FRPE material parameters with structure and composition of cartilage

The multivariable regression analyses of mechanical parameters and structure/composition at various tissue depths (0-5 %, 0-15, 0-20 %, 0-50%):

Table S1: The linear regression coefficients of structural and compositional properties (0-5% and 0-15 % of the tissue thickness) with the mechanical material parameters (*** p< 0.001, ** p < 0.01, * p < 0.05)

Dependent variable	Standardized regression coefficient β							
	Tissue thickness at 0-5%				Tissue thickness at 0-15%			
	Adjusted R^2	PG content	Collagen orientation angle	Collagen content	Adjusted R^2	PG content	Collagen orientation angle	Collagen content
E_f^0	0.30	**0.37***	0.31	0.08	0.43	**0.49****	-0.34	0.11
E_f^ε	0.20	-0.33	-0.31	0.09	0.02	-0.12	-0.29	0.15
E_{nf}	0.23	**0.41***	-0.32	0.07	0.41	**0.57****	-0.27	0.18
k_0	0.10	-0.27	0.02	0.17	0.02	-0.32	0.07	0.17
M	0.10	-0.06	-0.31	0.04	0.08	-0.12	-0.18	0.15
E_{eq}	0.48	**0.51****	**-0.37***	0.02	0.60	**0.69*****	**-0.31***	0.09
E_{inst}^0	0.38	**0.43***	**-0.38***	-0.02	0.46	**0.56****	**-0.37***	0.05
E_{inst}^ε	0.11	-0.17	-0.37	0.16	0.13	0.05	-0.39	0.19
E_{dyn}	0.18 ~ 0.22	0.21 ~ 0.22	**-0.43* ~ -0.47***	0.04 ~ 0.06	0.38 ~ 0.42	**0.45** ~ 0.43****	**-0.41* ~ -0.44****	0.12 ~ 0.13
$\theta_{0.005}$	0.40	**-0.40***	0.04	**0.55****	0.36	**-0.36***	0.11	**0.55****
θ_i	0.09 ~ 0.17	-0.07 ~ -0.17	0.36 ~ 0.41	0.23 ~ 0.34	0.07 ~ 0.28	-0.16 ~ 0.24	0.36 ~ **0.41***	0.23 ~ 0.34

E_f^0: initial fibril network modulus, E_f^ε: strain-dependent fibril network modulus, E_{nf}: non-fibrillar matrix modulus, k_0: initial permeability, M: permeability strain-dependency coefficient, E_{eq}: equilibrium modulus, E_{inst}^0: initial instantaneous modulus, E_{inst}^ε: strain-dependent instantaneous modulus, E_{dyn}: dynamic moduli at frequencies 0.005, 0.05, 0.1, 0.25, 0.5, 0.625, 0.833 and 1 Hz, $\theta_{0.005}$: phase difference at 0.005 Hz, θ_i: phase differences at frequencies 0.05, 0.1, 0.25, 0.5, 0.625, 0.833 and 1 Hz

Table S2: The linear regression coefficients of structural and compositional properties (0-20% and 0-50 % of the tissue thickness) with the mechanical material parameters (*** p< 0.001, ** p < 0.01, * p < 0.05)

Dependent variable	Standardized regression coefficient β							
	Tissue thickness at 0-20%				Tissue thickness at 0-50%			
	Adjusted R^2	PG content	Collagen orientation angle	Collagen content	Adjusted R^2	PG content	Collagen orientation angle	Collagen content
E_f^0	0.45	**0.52****	**-0.35***	0.13	0.38	**0.42***	**-0.35***	0.26
E_f^ε	0.01	-0.01	0.30	0.14	0.07	0.26	-0.25	0.19
E_{nf}	0.48	**0.61*****	-0.25	0.15	0.37	**0.54****	-0.22	0.24
k_0	0.03	-0.34	0.05	0.16	0.03	-0.34	-0.06	0.17
M	0.01	-0.13	-0.17	0.20	0.19	0.23	-0.29	**0.38***
E_{eq}	0.63	**0.74*****	**-0.27***	0.13	0.58	**0.70*****	-0.25	0-25
E_{inst}^0	0.45	**0.60*****	**-.035***	0.09	0.35	**0.51****	**-0.35***	0.16
E_{inst}^ε	0.18	0.16	**-0.42***	0.19	0.28	0.32	**-0.37***	0.32
E_{dyn}	0.46 ~ 0.54	**0.53**** ~ **0.54****	**-0.41*** ~ **-0.44****	0.14 ~ 0.15	0.46 ~ 0.48	**0.53**** ~ **0.54****	**-0.40*** ~ **-0.44****	0.14 ~ 0.15
$\theta_{0.005}$	0.30	**-0.36***	0.01	**0.52****	0.20	-0.24	0.08	**0.49***
θ_i	0.19 ~ 0.29	-0.25 ~ **-0.38***	0.36 ~ **0.47***	0.21 ~ 0.32	0.29 ~ 0.36	-0.34 ~ **-0.44***	0.26 ~ **0.34***	0.25 ~ 0.30

E_f^0: initial fibril network modulus, E_f^ε: strain-dependent fibril network modulus, E_{nf}: non-fibrillar matrix modulus, k_0: initial permeability, M: permeability strain-dependency coefficient, E_{eq}: equilibrium modulus, E_{inst}^0: initial instantaneous modulus, E_{inst}^ε: strain-dependent instantaneous modulus, E_{dyn}: dynamic moduli at frequencies 0.005, 0.05, 0.1, 0.25, 0.5, 0.625, 0.833 and 1 Hz, $\theta_{0.005}$: phase difference at 0.005 Hz, θ_i: phase differences at frequencies 0.05, 0.1, 0.25, 0.5, 0.625, 0.833 and 1 Hz

The multivariable regression analyses of phase differences (θ_i) and structure/composition:

Table S3: The linear regression coefficients of phase differences and structural and compositional properties of the superficial (0-10% of the tissue thickness) and whole tissue (*** p< 0.001, ** p < 0.01, * p < 0.05)

Dependent variable	Standardized regression coefficient β							
	Superficial layer (0-10%)				Whole tissue (0-100%)			
	Adjusted R^2	PG content	Collagen orientation angle	Collagen content	Adjusted R^2	PG content	Collagen orientation angle	Collagen content
$\theta_{0.05}$	0.19	-0.24	**0.38***	0.35	0.43	**-0.51****	0.26	0.37
$\theta_{0.1}$	0.12	-0.16	**0.41***	0.23	0.39	**-0.53****	0.27	0.29
$\theta_{0.25}$	0.12	-0.15	0.36	0.22	0.40	**-0.53****	0.24	0.32
$\theta_{0.5}$	0.12	-0.09	**0.37***	0.22	0.35	**-0.48****	0.27	0.32
$\theta_{0.625}$	0.10	0.17	0.30	0.17	0.44	**-0.58****	0.22	0.31
$\theta_{0.833}$	0.24	-0.02	**0.41***	0.19	0.49	**-0.59*****	0.31	0.29
$\theta_{1.0}$	0.18	-0.14	**0.41***	0.18	0.46	**-0.58****	0.29	0.29

The statistical power analyses for group-wise comparisons:

Table S4: The power analyses for statistically significant linear mixed model parameters

Parameter	Statistical significance p-value	Observed power †
E_{f}^{0} (initial fibril network modulus)	0.001	0.975
E_{inst}^{0} (initial instantaneous modulus)	0.00002	0.999
E_{nf} (non-fibrillar matrix modulus)	0.0002	0.991
E_{eq} (equilibrium modulus)	0.000003	0.999
Superficial proteoglycan content (0-10% tissue thickness)	0.001	0.967
Superficial collagen orientation angle (0-10% tissue thickness)	0.002	0.938

† Observed power calculated using alpha = 0.05

Table S5: The power analyses for group-wise comparisons (i.e. healthy versus OA groups) in statistically significant linear mixed models

Parameter	Post-Hoc test	Statistical significance p-value	Observed power †
E_{f}^{0} (initial fibril network modulus)	**Healthy Vs. Early OA**	**0.009**	**0.47**
	**Healthy Vs. Advanced OA**	**0.004**	**0.70**
	Early OA Vs. Advanced OA	0.99	0.15
E_{inst}^{0} (initial instantaneous modulus)	**Healthy Vs. Early OA**	**0.0002**	**0.77**
	**Healthy Vs. Advanced OA**	**0.00001**	**0.84**
	Early OA Vs. Advanced OA	0.99	0.12
E_{nf} (non-fibrillar matrix modulus)	**Healthy Vs. Early OA**	**0.003**	**0.54**
	**Healthy Vs. Advanced OA**	**0.0001**	**0.69**
	Early OA Vs. Advanced OA	0.99	0.55
E_{eq} (equilibrium modulus)	**Healthy Vs. Early OA**	**0.000002**	**0.82**
	**Healthy Vs. Advanced OA**	**0.0003**	**0.97**
	Early OA Vs. Advanced OA	0.33	0.56
Superficial proteoglycan content (0-10% tissue thickness)	**Healthy Vs. Early OA**	**0.003**	**0.68**
	**Healthy Vs. Advanced OA**	**0.001**	**0.61**
	Early OA Vs. Advanced OA	0.99	0.12
Superficial collagen orientation angle (0-10% tissue thickness)	Healthy Vs. Early OA	0.07	0.77
	**Healthy Vs. Advanced OA**	**0.0003**	**0.99**
	Early OA Vs. Advanced OA	0.165	0.79

† Observed power calculated using alpha = 0.05

Discussion

The frequency regime used in the study ranged from 0.005 to 1 Hz, but we also collected data at 2 Hz. This frequency range is physiologically relevant as the frequencies of loading on cartilage for daily routine activities range from very low frequencies (representing e.g. standing with an occasional shift of weight) to 3.5 Hz for running [11], with a typical walking frequency of ~ 0.4-2 Hz [12]. However, as the contact between the sample and indenter was not appropriate during the unloading phase at frequencies over 1 Hz (i.e. the reaction force measured by the load-cell dropped to zero), we were not able to obtain reliable data from higher frequencies. Moreover, we observed that the phase difference and dynamic modulus reached a plateau at around 0.1 Hz, suggesting that the dynamic properties would be similar at frequencies higher than 1 Hz. In fact, we checked the results just from the loading phase of the sinusoidal data, and the dynamic modulus at 2 Hz was the same as that at 1 Hz. We analyzed also fluid pressure as a function of frequency by using a fibril-reinforced poroelastic model of a healthy sample (Figure S3). It follows the change in dynamic modulus.

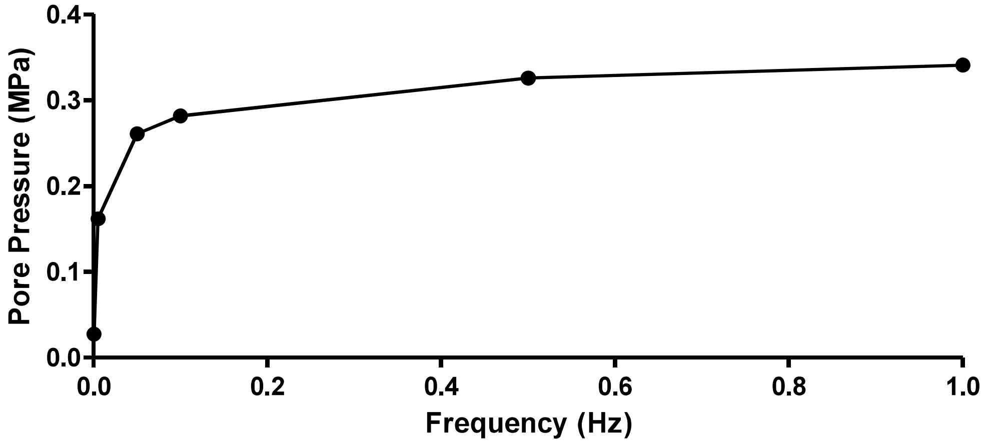

Figure S3: Fluid pressure as a function of frequency as predicted by the fibril-reinforced poroelastic FE model (material parameters from a healthy cartilage sample).

In our previous study [3], we observed smaller strain-dependent fibril network modulus in the OARSI grade 0 group than that in the rest of the OARSI grade groups. We presumed that this is due to the more homogenous structure in the OARSI 0 samples. Based on the findings of this study, the negative rank correlation between the collagen orientation angle and the strain-dependent fibril network modulus (and similarly the strain-dependent instantaneous modulus) suggest that collagen disorganization is a

dominant factor to modulate the strain-dependent fibril network modulus. In the superficial layer of the healthy samples, the collagen fibrils were more tangentially oriented (more homogenous superficial structure). In the OA groups, however, the collagen disorganization leads to structural nonhomogeneity. With a greater degree of the collagen disorganization, especially at the superficial and middle/transient layers of OA samples compared to healthy ones, initially, the collagen fibrils resist less tensile stresses as they are progressively recruited (which leads to higher strain-dependent fibril network modulus). However, in the homogenous tissue, the fibrils are likely to be recruited once the tissue is compressed (which is reflected in greater initial fibril network modulus). Therefore, the strain-dependent fibril network modulus was lower in the OARSI 0 grade group.

References

1. Deneweth, J. M., K. E. Newman, S. M. Sylvia, S. G. McLean, and E. M. Arruda. Heterogeneity of tibial plateau cartilage in response to a physiological compressive strain rate. *J. Orthop. Res.* 31:370–375, 2013.

2. DiSilvestro, M. R., and J.-K. F. Suh. A cross-validation of the biphasic poroviscoelastic model of articular cartilage in unconfined compression, indentation, and confined compression. *J. Biomech.* 34:519–525, 2001.

3. Ebrahimi, M., S. Ojanen, A. Mohammadi, M. A. Finnilä, A. Joukainen, H. Kröger, S. Saarakkala, R. K. Korhonen, and P. Tanska. Elastic, Viscoelastic and Fibril-Reinforced Poroelastic Material Properties of Healthy and Osteoarthritic Human Tibial Cartilage. *Ann. Biomed. Eng.* 47:953–966, 2019.

4. Julkunen, P., R. K. Korhonen, M. J. Nissi, and J. S. Jurvelin. Mechanical characterization of articular cartilage by combining magnetic resonance imaging and finite-element analysis—a potential functional imaging technique. *Phys. Med. Biol.* 53:2425, 2008.

5. Korhonen, R. K., M. S. Laasanen, J. Töyräs, J. Rieppo, J. Hirvonen, H. J. Helminen, and J. S. Jurvelin. Comparison of the equilibrium response of articular cartilage in unconfined compression, confined compression and indentation. *J. Biomech.* 35:903–909, 2002.

6. Kulmala, K. A. M., H. J. Pulkkinen, L. Rieppo, V. Tiitu, I. Kiviranta, A. Brünott,

H. Brommer, R. van Weeren, P. A. J. Brama, and M. T. Mikkola. Contrast-enhanced micro–computed tomography in evaluation of spontaneous repair of equine cartilage. *Cartilage* 3:235–244, 2012.

7. Lamela, M. J., F. Pelayo, A. Ramos, A. Fernández-Canteli, and E. Tanaka. Dynamic compressive properties of articular cartilages in the porcine temporomandibular joint. *J. Mech. Behav. Biomed. Mater.* 23:62–70, 2013.

8. Mäkelä, J. T. A., S. K. Han, W. Herzog, and R. K. Korhonen. Very early osteoarthritis changes sensitively fluid flow properties of articular cartilage. *J. Biomech.* 48:3369–3376, 2015.

9. Mäkelä, J. T. A., M. R. J. Huttu, and R. K. Korhonen. Structure–function relationships in osteoarthritic human hip joint articular cartilage. *Osteoarthr. Cartil.* 20:1268–1277, 2012.

10. Mäkelä, J. T. A., Z. S. Rezaeian, S. Mikkonen, R. Madden, S.-K. Han, J. S. Jurvelin, W. Herzog, and R. K. Korhonen. Site-dependent changes in structure and function of lapine articular cartilage 4 weeks after anterior cruciate ligament transection. *Osteoarthr. Cartil.* 22:869–878, 2014.

11. Nilsson, J., and A. Thorstensson. Adaptability in frequency and amplitude of leg movements during human locomotion at different speeds. *Acta Physiol. Scand.* 129:107–114, 1987.

12. Öberg, T., A. Karsznia, and K. Öberg. Basic gait parameters: reference data for normal subjects, 10-79 years of age. *J. Rehabil. Res. Dev.* 30:210, 1993.

13. Tanaka, E., E. Yamano, D. A. Dalla-Bona, M. Watanabe, T. Inubushi, M. Shirakura, R. Sano, K. Takahashi, T. van Eijden, and K. Tanne. Dynamic compressive properties of the mandibular condylar cartilage. *J. Dent. Res.* 85:571–575, 2006.

14. Torniainen, J., A. Ristaniemi, J. K. Sarin, S. Mikkonen, I. O. Afara, L. Stenroth, R. K. Korhonen, and J. Töyräs. Near Infrared Spectroscopic Evaluation of Ligament and Tendon Biomechanical Properties. *Ann. Biomed. Eng.* 47:213–222, 2019.
